# Designing and measuring the progress and impact of health research capacity strengthening initiatives

**DOI:** 10.1186/1753-6561-9-S10-S9

**Published:** 2015-12-18

**Authors:** Imelda Bates

**Affiliations:** 1 Professor Imelda Bates is affiliated with the Liverpool School of Tropical Medicine (LSTM), Liverpool, UK

## Abstract

Strengthening capacity in poorer countries to generate multi-disciplinary health research and to utilise research findings, is one of the most effective ways of advancing the countries' health and development. This paper explores current knowledge about how to design health research capacity strengthening (RCS) programmes and how to measure their progress and impact. It describes a systematic, evidence-based approach for designing such programmes and highlights some of the key challenges that will be faced in the next 10 years. These include designing and implementing common frameworks to facilitate comparisons among capacity strengthening projects, and developing monitoring indicators that can capture their interactions with knowledge users and their impact on changes in health systems.

## Measuring and learning about health research capacity strengthening for development

Strengthening health research capacity in low and middle income countries (LMICs) is one of the most effective ways of advancing their health and development. Over the next 10-20 years the current trend towards more emphasis on capacity strengthening within health research projects is likely to accelerate. This is because lack of capacity to utilise research findings, to integrate them into health systems and embed them in institutions, is a major bottleneck in our ability to maximize the benefits of research for development.

**Definition of research capacity strengthening** - *By health RCS we mean a ‘process of individual and institutional development which leads to higher levels of skills and greater ability to perform useful research.’*

Finding the best ways to do capacity strengthening and to measure its effectiveness requires a much better understanding of the processes involved than we have at present. It will involve critically reviewing and sharing lessons about what works and what doesn't work, and using much more robust ways to measure the impact of research capacity strengthening (RCS) efforts. Common frameworks to guide health RCS efforts will be needed[1] to facilitate comparison among different projects and for real-time lesson learning (for examples see table 1).

**Table 1:** Example of using a common framework based on ‘phases’ of progress to systematically compare case studies of sustainable capacity strengthening programmes[11]

Project phase	Indicators of capacity strengthening progress (outcomes/outputs and approximate date achieved)			
	**Case study 1 KATH, Ghanaian teaching hospital**	**Case study 2 Kenyan NGO LVCT**	**Case study 3 Malawian Research Unit REACH Trust**	**Case Study 4 DRC Research and Training. IPASC**

Aim of original project	To promote generation of local evidence to improve health care	To scale up access to HIV counselling and testing in primary health care centres	To develop evidence on equity, poverty and access to TB services in Malawi.	To understand the health needs of the community and develop context specific responses

Aim of capacity building component	Improve ability of teaching hospital to sustainably deliver and manage research skills course to UK standards without external resources	Improve ability of health care facility teams to deliver quality assured HIV counselling and testing and contribute to research findings	To build research skills in equity analysis and multi-method research to develop policy-relevant research	To provide training in community health grounded in context for different cadres

**Capacity building activities**				

Awareness phase “planning, awareness raising”	LSTM and KATH/KNUST jointly commit funds to improve capacity for conducting and using research Framework for monitoring progress developed	High HIV care burden in health care facilities with little knowledge of HIV status Recognition of lack of evidence about feasibility of this approach	Recognised need for operational research to guide NTP priorities Collaboration between NTP, LSTM and University of Malawi Obtained project funding	Recognised need for research, training and infrastructure development appropriate for rural, conflict/post-conflict DRC.
*Timing (months from start)*	*Started 2002 0-12*	*Started 2001 0-18*	*Started 1999 0-1*	*Started 1992 0-36*

Experiential phase “start up, testing”	UK off-site Diploma (DPDM) established in Ghana for all KATH health professionals Institutional research support services increased (e.g. internet access, research office established, earmarked local project funds); creation of faculty team	33 primary health facilities provide counselling and testing Kenyan NGO (LVCT) established for technical assistance to government to achieve scale up Research findings inform Kenyan guidelines and training	Studies conducted and fed into NTP policy and practice through Technical Working Groups First round of staff get Masters by Research from University of Malawi	IPASC is launched First graduates get degrees IPASC staff trained at LSTM on masters and PhD programmes
*Timing (months from start)*	*9-36*	*12-36*	*12-24*	*24-108*

Expansion phase “scale up, innovation”	Sustainable funding from MoH KATH fund quality assurance by LSTM Faculty for DPDM established with dedicated administration team First paper published by DPDM graduate	NGO expands to incorporate other post rape care, services for the disabled and for vulnerable groups Range of donors broadened and core funds increased First papers published	New staff recruited and research portfolio broadens to include HIV. Range of donors broadens and includes MoH funding Malawian director appointed; technical assistance from LSTM Malawian first author papers published	New courses established Range of donors broadened Became part of the EQUINET network Obtained funding to expand research DRC first author paper published
	24-60	30-72	40-60	108-192

*Timing (months from start)*				

Consolidation phase “sustainability, autonomy”	DPDM run entirely by KATH tutors; LSTM monitor quality Research results fed into clinical audit cycles Grants obtained with local researchers as lead DPDM expanded to second institution Further publications from DPDM graduates	Kenyan-run NGO with links to LSTM through Board of Trustees and collaborative research projects Over 500 HIV counselling and testing sites established Programme twinned with other countries in SSA. Research findings incorporated in international policy	REACH Trust - Independent Malawian research Trust established with Board of Trustees and Malawian Director Diverse funding and research portfolio. Ongoing advocacy with MoH and policy contributions.	Fully DRC run with global links to funders and academics
*Timing (months from start)*	*48-84*	*6-120*	*60-120*	*12-192*

Health RCS takes many forms and operates at three different levels, often simultaneously – *individual* (e.g. PhD training), *institutional* (e.g. establishing research governance systems) and *national/international* (e.g. international collaborations). The complexity and heterogeneity of RCS initiatives[2] makes it difficult to evaluate their effectiveness.

Our Capacity Research Unit at LSTM does research to inform better design and measurement of RCS programmes. From our work with health research funders, research institutions and national research systems, and through our own experience of managing capacity strengthening programs in LMICs we have developed an evidence-based 5-step approach to designing RCS programmes which is applicable to diverse contexts. We have collated information about different approaches to RCS and summarized what works best in which type of context and why. We have analysed evaluators' reports to bring together a comprehensive list of indicators that have been used to measure RCS processes and impact.

Our research has inevitably unearthed some critical priorities, challenges and tensions that need to be addressed over the next 10-20 years to ensure we make significant progress in bridging the gap between research generation and population health and development in LMICs. Integration of different research disciplines, as well as enhanced research capacity, is essential to make a significant impact on strengthening health systems and to support effective policies and decision-making. In this paper we outline some of the key findings of our research and highlight what has emerged from our research as priorities for the next 10 years.

## Designing RCS programmes: 5-step approach

Evidence to guide the design and monitoring of RCS efforts in LMICs is limited and formulating a general approach is complex because each programme is unique. Capacity strengthening programmes usually start off with a ‘needs assessment’ but by doing this, two critical steps are missed out – defining the expected outcomes and identifying the capacity needed to achieve the outcomes. Both these steps are important in developing a robust and comprehensive action plan and in promoting ownership and sustaining change. Using published evidence, including literature on organisational management, we have developed a five step pathway for designing and evaluating RCS programmes and tested it in a variety of contexts in Africa[3-5].

The pathway involves:

a) defining the goal of the capacity strengthening effort,

b) describing the optimal capacity needed to achieve the goal,

c) determining the existing capacity gaps compared to the optimum,

d) devising an action plan to fill the gaps and associated indicators of change, and

e) adapting the plan and indicators as the programme matures.

The pathway starts with a clear goal and objectives, and it make the capacity required to achieve the goal explicit. We derive a description of the optimal capacity needed to achieve the goal from a comprehensive search of the literature and include inputs by international experts relevant to the goal of the RCS, and a ‘reality check’ by LMIC partners. The unique aspect of this 5-step approach is the production of this evidence-based ‘benchmark’ (i.e. definition of optimal capacity needed to achieve the goal) which is designed specifically for each project and against which existing capacity is compared. This approach differs from most needs assessments of research capacity which do not use a rigorous benchmark and therefore are not able to provide reassurance that significant areas have not been overlooked.

Using the optimal capacity ‘benchmark’ we identify capacity gaps and develop the action plan to fill the gaps in collaboration with LMIC partners. The plan is designed to be flexible enough to generate and utilise ongoing learning; strategies for promoting sustainability are incorporated from the outset. The plan takes into account not only technical, managerial and financial processes within an organisation, but also the individuals who work in the organisation and the wider system within which the organisation operates and is financed and managed. Interestingly we have consistently found when using this five-step approach in practice, that 60-70% of activities in the plan are ‘no cost’. For example, establishing a new committee, drafting protocols and strategies, or providing in-house training.

### Five step pathway for designing health research capacity strengthening programmes

**1. Define the goal of the capacity strengthening project**.

This necessitates harmonising the expectations and objectives of the most critical stakeholders including developing country partners, people involved in ensuring sustainability of the activities in the long term and the funding organisation.

**2. Describe the required capacity needed to achieve the goal**.

This will require a search for the best evidence to describe the ‘optimal’ capacity collated from, for example, peer-reviewed published papers or expert groups, including evidence from outside the health sector.

**3. Determine the existing capacity and identify any gaps compared to the required capacity**.

The evidence from step two is formatted into a set of qualitative and quantitative data collection tools to identify existing capacity and capacity gaps. Data is collected from stakeholders with different perspectives; discrepancies are highlighted and resolved through further discussion.

**4. Devise and implement an action plan to fill the gaps**.

The prioritised list of capacity gaps is transformed into an action plan which includes objectives, activities, deliverables and monitoring indicators, and measures to facilitate sustainability

**5. Learn through doing; adapt the plan and indicators regularly**.

Results from experimentation and learning, and regular discussions with those responsible for monitoring progress are used to refine the plan. Progress indicators become more sophisticated as the programme matures and capacity is strengthened

### 10 year perspective for designing RCS programmes to enable measurements of progress and impact and allow comparisons

*In order to facilitate measurements of progress and impact of individual RCS programmes, and to allow comparisons between programmes, much more effort needs to be made to develop and use common principles, and if possible, a generic approach, for designing RCS programmes*.

## Investing in learning from good quality RCS evaluations

Understanding what indicators or metrics can be used for health RCS and their appropriateness, or not, in different contexts, is critical to monitor the performance of RCS programmes, to measure achievement and to demonstrate accountability. Ideally RCS funders would like a few common measurable, reliable indicators for measuring RCS but they also need to demonstrate project impact on, for example, policies and poverty, for which direct attribution is difficult to prove. Our research has shown that the majority of evaluations of health RCS meet very few of the generally accepted quality standards. Most of the evaluations we have reviewed were retrospective so they lack comparative baseline data, and the purpose and indicators for outcomes and impact were often not stipulated.

We were unable to find information in the published literature about indicators for RCS that were SMART (Specific, Measurable, Attainable, Realistic and Time Bound). We therefore collaborated with a group of global health research funders and collated all the indicators we could find in their evaluation reports of RCS evaluations[6]. We extracted the indicators into a matrix across individual, institutional and national/international levels. Critical to making sense of RCS outcomes is the need to be explicit about the pathway by which change is to be brought about (i.e. the theory of change) and to use indicators that reflect different stages on the pathway. We therefore synthesized indicators across potential impact pathways (activities to outputs to outcomes) and iteratively verified our findings with key health RCS evaluation stakeholders. The indicators we identified were primarily of activities, outputs or outcomes with a strong bias towards the more easy-to-measure individual level indicators. There was very little information on the inter-relationships along a ‘change pathway’ so measurements of progress in developing research capacity over time were scarce.

## Define the underlying ‘pathway to change’

RCS is a complex process with a long intervention pathway and examples of good quality RCS evaluations are hard to find. Such evaluations involve theory-driven evaluative thinking, identification of underlying assumptions and an explicit rationale for any actions[7]. It is important to consider long-term impact across different levels throughout the whole project cycle using clear conceptual frameworks, multiple data sources and valid standards to enhance quality.

RCS evaluations should incorporate a theory of change as this helps to identify impact trajectories, strengthen evaluation rigour, foster assessment of generalisability to other contexts, and guide influence on policy and practice. However, most evaluations currently do not have time and resources to incorporate theory-informed indicators of impact and sustainability, or to collect data against these indicators. This means opportunities to enhance knowledge and learning among funders and funding recipients about how to improve planning, monitoring and evaluation of health RCS initiatives are being missed[8].

## Sharing lessons among research funders

Some funder groups (e.g. UK Collaborative on Development Sciences, the ESSENCE on Health Research Initiative) are now coming together to share experiences and learn from health RCS initiatives but they are hampered by a lack of common approaches and of systematic methods for documenting RCS. Ultimately, timely sharing information about RCS will help to avoid repeating mistakes, and enhances knowledge about how to do better health RCS. Our research highlighted some key challenges concerning learning from RCS efforts that will need to be addressed in future. Health RCS activities are often ‘bolted on’ to research projects but expectations that researchers themselves will spend a significant amount of time documenting and using learning to improve the RCS activities at the same time as delivering their primary research, may be unrealistic. Research funders face a trade-off between their need to show accountability and value for money, and the extent to which they should invest in facilitating and sharing learning.

## Engagement of stakeholders in evaluations of RCS

Our research suggests that funding recipients and other stakeholders should be actively involved in all stages of the RCS evaluation process as this ‘empowerment’ makes RCS initiatives more likely to become self-sustaining[9]. It also helps to detect and correct problems early, makes the decisions underlying the evaluations more visible, and encourages sharing and use of the evaluation results. Stakeholders such as funding recipients have greater in-depth knowledge about the project and its context compared to external evaluators, which is vital for solving problems and sustainability. However there is a tension between this approach, which takes time and needs an additional, ear-marked budget, and the traditional summative model of evaluation carried out by an independent external team, which many funders' perceive as better value for money, for demonstrating accountability and for producing rapid results[10].

**10 year perspective on using learning from research to design more effective RCS programmes**

### 10 year perspective for measuring RCS progress and impact

*Tools and methods for measuring RCS are in their infancy. There is huge scope for progress in this area over the next decade particularly in defining better indicator measurement properties and in developing indicators that encompass relationships with knowledge users. Indicators need to be more sophisticated so they can provide information not only on knowledge production and capacity development at individual level, but also on changes in health system policies, programs and practices. Greater attention to evaluation design, prospective indicator measurement, and systematic linkage of indicators in keeping with theories of change could provide more robust evidence on outcomes of health RCS. The impact and expected outcomes of RCS programmes should be defined. A paradigm shift will be needed to accept that the focus should be on assessing the ‘contribution’ of a programme, to this impact goal rather than trying to directly attribute impact to an individual programme*.

*In future each RCS project should develop comprehensive, prospective system for health RCS evaluation. Donors and policy makers could develop supporting guidance, tools or training for leading or participating in health RCS evaluations. Adequate funding will need to be committed to ensure high quality and meaningful evaluations of health RCS projects. To avoid the tensions faced by researchers and funders in prioritizing learning from RCS efforts, specialized evaluation teams could work closely with the researchers and funders, to take responsibility for the learning aspects of evaluations (i.e. to undertake ‘developmental’ evaluations)*.

### How to minimize future tensions in designing and measuring RCS efforts

Funders of health RCS initiatives and those involved in determining policies concerning RCS can play a key role in minimizing tensions that threaten the effectiveness of RCS efforts. They should:

1. Promote a critical debate on the tensions inherent in evaluation of health RCS.

2. Develop supporting guidance, tools and training for leading and participating in health RCS evaluations.

3. Allocate adequate funding to the evaluation of health RCS projects.

4. Systematically document lessons learned in M&E of health RCS projects and engage funders, implementers and evaluators in an effective learning process.

5. Develop a community of practice to share lessons and experiences and consider joint ways forward.

## Competing interests

The author declares that they have no competing interests.

## Abbreviations

DPDM: Diploma in Project Design and Management; EQUINET: The Network on Equity in Health in Southern Africa; IPASC: Institut Panafricain de Santé Communautaire; KATH: Komfo Anokye Teaching Hospital; KNUST: Kwame Nkrumah University of Science and Technology; LSTM: Liverpool School of Tropical Medicine; LVCT: Liverpool VCT, Treatment and Care; MoH: Ministry of Health; NGO: Non-governmental organisation; NTP: Malawi National Tuberculosis Control Programme; REACH: Research for Equity and Community Health Trust

## Declarations

This article has been published as part of *BMC Proceedings* Volume 9 Supplement 10, 2015: The 2014 CAHRD (Collaboration for Applied Health Research & Delivery) Consultation. The full contents of the supplement are available online at http://www.biomedcentral.com/bmcproc/supplements/9/S10.

## References

[B1] ESSENCE - TDR Seven principles for strengthening research capacity in low- and middle-income countries: simple ideas in a complex world. Good practice document series WHO reference number: TDR/ESSENCE/2.14. 2014 36 pp. http://www.who.int/tdr/publications/Essence_report2014_OK.pdf?ua=1 (accessed September 2015)

[B2] Ager A Zarowsky C Balancing the personal, local, institutional, and global: multiple case study and multidimensional scaling analysis of African experiences in addressing complexity and political economy in health research capacity strengthening Health Res Policy Syst 2015 doi:10.1186/1478-4505-13-5 10.1186/1478-4505-13-5 PMC4325946 25595847

[B3] Bates I Boyd A Smith H Cole DC A practical and systematic approach to organisational capacity strengthening for research in the health sector in Africa Health Research Policy and Systems 2014 12 11 24581148 10.1186/1478-4505-12-11 PMC3975897

[B4] Njelesani J Dacombe R Palmer T Smith H Koudou B A Systematic Approach to Capacity Strengthening of Laboratory Systems for Control of Neglected Tropical Diseases in Ghana, Kenya, Malawi and Sri Lanka PLoS Negl Trop Dis 2014 8 3 e2736 24603407 10.1371/journal.pntd.0002736 PMC3945753

[B5] Bates I Phillips R Martin-Peprah R Kibiki G Gaye O Phiri K Tagbor H Purnell S Assessing and strengthening African universities' capacity for doctoral programmes PLOS Medicine 2011 8 9 e1001068 21931538 10.1371/journal.pmed.1001068 PMC3172246

[B6] Cole DCC Boyd A Aslanyan G Bates I Indicators for tracking programmes to strengthen health research capacity in lower- and middle-income countries: a qualitative synthesis Health Research Policy and Systems 2014 12 17 24725961 10.1186/1478-4505-12-17 PMC3999501

[B7] Vogel I Review of the use of ‘Theory of Change’ in international development 2012 https://www.gov.uk/government/news/dfid-research-review-of-the-use-of-theory-of-change-in-international-development (accessed September 2015)

[B8] Boyd A Cole DC Dan-Bi C Aslanyan G Bates I Frameworks for evaluating health research capacity strengthening: A qualitative study Health Research Policy and Systems 2013 11 46 24330628 10.1186/1478-4505-11-46 PMC3878679

[B9] Ghaffar DA Jsselmuiden C Zicker F Changing mindsets: research capacity strengthening in low-and middle-income countries 2008 http://wwwlive.who.int/entity/tdr/publications/documents/changing_mindsets.pdf. (accessed September 2015)

[B10] Bates I Boyd A Aslanyan G Cole DC Tackling the tensions in evaluating capacity strengthening for health research in low- and middle-income countries Health Policy and Planning 2014 Apr 8. [Epub ahead of print] 10.1093/heapol/czu016 PMC4353897 24717984

[B11] Bates I Taegtmeyer M Squire SB Ansong D Nhlema-Simwaka B Baba A Theobald S Indicators of sustainable capacity building for health research: analysis of four African case studies Health Research Policy and Systems 2011 9 1 14 21443780 10.1186/1478-4505-9-14 PMC3078899

